# Knowledge and its factors associated towards the prevention of COVID-19 among pregnant women in Ethiopia

**DOI:** 10.4314/ahs.v22i4.64

**Published:** 2022-12

**Authors:** Muche Argaw Eniyew, Yibeltal Mesfin, Shegaw GezeTenaw, Bitew Tefera

**Affiliations:** 1 Department of Midwifery, College of Medicine and Health science, Wolkite University, Wolkite, Ethiopia; 2 Department of Nursing, College of Medicine and Health science, Wolkite University, Wolkite, Ethiopia

**Keywords:** COVID-19, pregnant women, knowledge, prevention

## Abstract

**Background:**

COVID-19 is caused by a single-stranded novel coronavirus that severely affects the respiratory system. The first human COVID-19 was reported in Wuhan city, China, in December 2019. Closing the gap and enhancing knowledge of pregnant women on COVID-19 prevention methods is crucial. However, the studies conducted in Ethiopia were inconsistent and non-conclusive. So, this review aimed to estimate the pooled knowledge prevalence on prevention of COVID 19 and factors associated among pregnant women in Ethiopia.

**Methods:**

The data were extracted based on the Preferred Reporting Items for Systematic Reviews and Meta-Analyses guidelines. We accessed studies through electronic web-based search from PubMed, Cochrane Library, and Google Scholar. We did all statistical analyses using STATA version 14 software with a random-effects model.

**Results:**

Seven studies with 2,594 participants were included in this systematic review and the overall estimated status of assessment of knowledge towards prevention of COVID-19 among pregnant women in Ethiopia was 52.27% (31.60, 68.94). According to the region subgroup analysis, the highest ad the lowest estimated status of the knowledge is 85.34% in Jimma town and 19.01 in Metu town respectively in the Oromia region.

**Conclusion:**

This systemic review showed that only half of the pregnant women in Ethiopia had good knowledge about COVID-19, and urban residence was significantly associated with knowledge towards the prevention of COVID-19 among pregnant women in this review. So, the responsible body better strengthen their awareness creation among rural residents and old-age pregnant women.

## Background

Coronavirus disease (COVID-19) is an illness caused by a novel coronavirus, severe acute respiratory syndrome coronavirus 2(SARS-COV-2 formerly called 2019-nov), which was the first outbreak of respiratory illness cases in Wuhan City, Hubei province, China 1. WHO reported as COVID-19 was an outbreak of the disease first in Wuhan city, China, in December 2019 2.

The way of transmission of COVID-19 is through respiratory droplets, physical contacts, fecal-oral, and an incubation period of 2–14 days 3.

Globally the cases and death reported were 952,507 and 498,519 respectively from 30, December 2019, to August 2020. Where came to Africa 371,448 cases and 9480 deaths were reported 4.

In sub-Saharan Africa, COVID-19 is low reported, however, it is due to the capacity for detection and reporting. In this region most severely affected due to limited resources, low vaccine coverage, and poor awareness about prevention mechanisms 5.

However, overall risks of pregnancy in COVID-19 are low, pregnant women were increased severe illness due to their immunosuppression and physiologic changes during pregnancy

There was also a shared by the evidence pregnant women affected with the COVID-19 may be exposed to preterm birth. About 127 pregnant women were from February 2020 to June 2020 in Brazil were exposed to preterm due to COVID-19 6.

Pregnant women with pre-existing medical diseases, including DM, bacterial, viral infection, and obstetric complications such as preeclampsia, cesarean section, PROM, placenta previa, and PPH were highly susceptible to the COVID-19 virus. It is also strongly associated with adverse birth outcomes such as fetal distress, fetal tachycardia, low birth weight, neonatal asphyxia, and stillbirth 7. A pandemic disease of COVID-19 also increases the risk of perinatal anxiety, maternal depression, and domestic violence in pregnant women 8.

The government of Ethiopia implements various actions ranging from public health emergency response to lockdown^9^. However, Ethiopia has been severely affected by COVID 19. There have been 308,134 confirmed cases of COVID-19 with 4,675 deaths from May 2020, to September 1, 2021, and This prevalence is also a burden on pregnant women due to a low level of awareness and knowledge^10^. The study conducted in the southern part of Ethiopia assessing knowledge and its associated factors towards the prevention of COVID 19 is below half^11^. Closing the gap and enhancing knowledge of pregnant women on COVID-19 prevention methods is crucial. However, the studies conducted in Ethiopia were inconsistent and non-conclusive. So, the aims of this review were conducted to estimate the pooled assessment of knowledge towards prevention of COVID 19 and COVID-19 getting factors associated among pregnant women in Ethiopia.

## Methods

### Study design and searching procedure

This review aims to summarize the published articles on the knowledge, and associated factors of COVID-19 among pregnant women in Ethiopia. The search engine of this systematic review study was found in Hinari, Google Scholar, EMBASE, Scopus, and PubMed/midline. All articles until July 30/2021 were included in the review. We used the terms such as the prevalence of knowledge and its associated factors of the prevention of COVID-19 among pregnant women, towards the prevention of COVID-19 among pregnant women, and prevention of COVID-19, and its associated factors of pregnant women in Ethiopia. Finding the reference was already identified studies to retrieve additional articles were done. The study has taken place according to preferred reporting items for systematic reviews and meta-analysis (PRISMA).

### Eligibility criteria

All cross-sectional articles reported on the knowledge prevalence of the prevention COVID- 19 and its factors associated among pregnant women in Ethiopia published in the English language, which contain relevant outcomes, were included. Initially, we obtained 31 articles using a systematic search in the database. Two articles were removed due to duplications, then 16 articles were removed because their abstract, title and full texts were not complete or available. The remaining 6 articles were excluded after reading the full article due to did not report the outcome. Finally, 7 articles that met the inclusion criteria were included in the final review.

### Data extraction

The authors extracted the articles of eligible studies in the form data abstraction sheet. The way of extracted information on the name of the first author and year of publication, country/region, study designs, the number of the case, response rate, sample size, and their associated factors.

### Methodology of quality assessment

The National Institutes of Health (NIH) quality Assessment tool was used to determine the quality of the studies. Accordingly, each question was answered with “yes,” “no” or “cannot determine” “ not applicable” and “not reported”^12^.

### Data Processing and Analysis

We used STATA version14.1 software for the data analysis. To check potential publication biases funnel plot and Egger's test were running. To assess the Heterogeneity of the study Cochrane Q tests and I2 with corresponding value P were used. Hence, there was marked heterogeneity within studies; the random effect model was used to compute the pooled prevalence of assessment knowledge of COVID-19 among pregnant women. Furthermore, for the evidence of heterogeneity within studies subgroup analysis was computed. Moreover, the estimated pooled prevalence rate was reported on a 95% confidence interval (CI), and a P-value < 0.05 was considered statistically significant. The odds ratio was calculated to measure the association between the outcome variable and their factors.

## Results

### Search results

**Figure 1:**
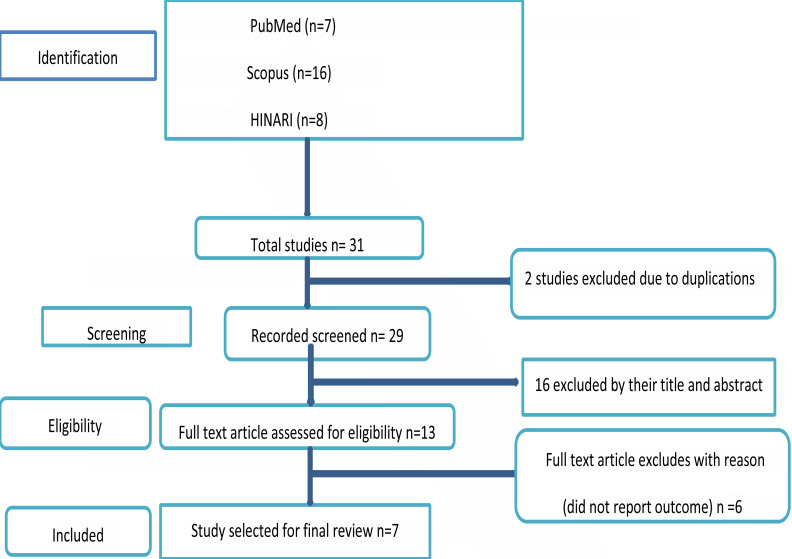
Diagram of the study selection for systematic and Meta-analysis of knowledge COVID-19 among pregnant women in Ethiopia

Initially, 31 publications were obtained from three databases (PubMed, HINARI, and Scopus). After that remove two studies due to duplicates, and the remaining articles were 29. We have also excluded 16 articles due to their titles and abstracts. As finally only13 articles were subjected to full fill-text review. In the end, seven articles that fulfilled the eligibility criteria were included in the systemic review (Fig. 1).

### Characteristics of identified studies

This review included seven studies in the final. All study conducted until 2021 was included. It represented three regions in this systemic review. Three studies from the Oromia Region, three studies from the Amhara region, and one study from the south region in Ethiopia were included (Table 1).

**Table 1:** Descriptive summary of seven studies in systemic and meta-analysis of knowledge towards prevention of COVID-19 and its associated factors among pregnant women in Ethiopia

Name author	Year of publication	study area, Region	study design	cases	sample size	Response rate
Dule et al	2020	Oromia	CS	73	384	87
Fikadu et al	2020	Southern	CS	306	403	76
Tadess et al	2020	Amahara	CS	114	389	29
Aboma et al	2021	Oromia	CS	198	232	83
W/mariam et al	2020	Amahara	CS	211	422	55
Janakiraman et al	2021	Amahara	CS	138	349	39.5
Besh et al	2021	Oromia	CS	219	415	90.6

CS, reference to cross -sectional

### Assessment of Knowledge towards prevention of COVID-19 among pregnant women

**Figure 2:**
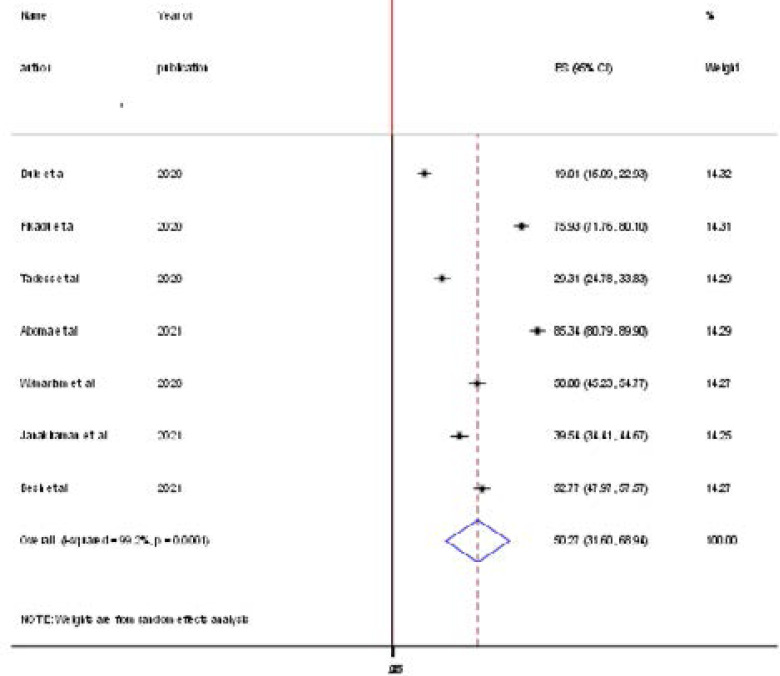
Forest plot in the pooled prevalence of knowledge to the prevention of COVID- 19 among pregnant women in Ethiopia

The lowest prevalence of knowledge towards prevention of COVID-19 was 19% observed study conducted in Oromia Region in menu town and the maximum prevalence also 85% study conducted in Jimma town in Oromia Region^13^, ^14^. The pooled national prevalence of knowledge on the prevention of COVID-19 was 50.27% (95% CI: 31.6, 68.9) based on the random effect analysis (figure 2).

### Sub-group analysis

**Figure 3:**
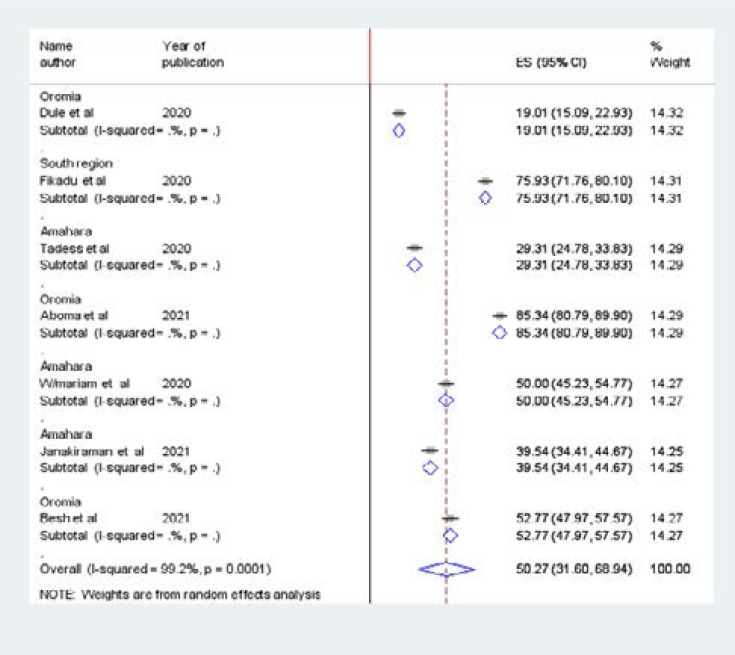
Forest plot showing the subgroup analysis of knowledge towards prevention of COVID-19 in Ethiopia by region

Subgroup analysis was done by region and the study population was calculated to compare assessment knowledge and its associated factors towards preventing COVID-19 among pregnant women across the region of the country. Accordingly, the highest prevalence was observed in Oromia Region, Jima zone 85.34% (80.79, 89.9) followed by the Amhara region 50% (45.23, 54.77). The lowest knowledge 19% (95% CI: 15.09, 22.99) was observed in Metu town, Oromia Region (fig.3).

### Heterogeneity and publication bias

**Figure 4:**
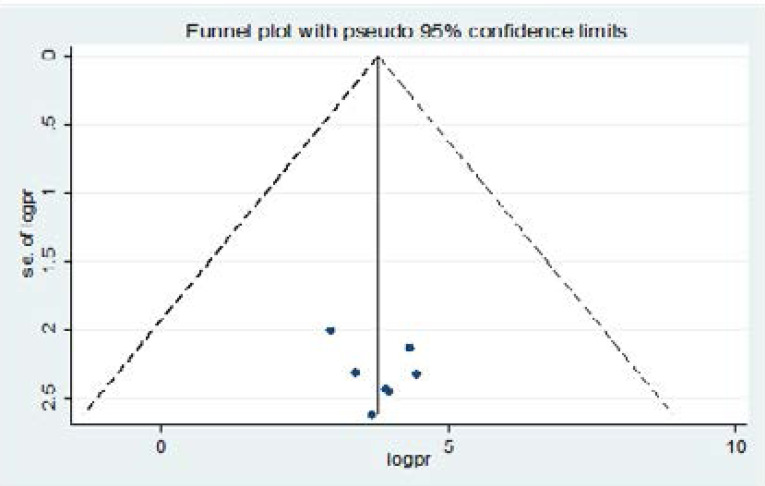
Funnel plot depicting publication bias of studies reporting knowledge and associated factors towards prevention of COVID-19 among pregnant women in Ethiopia, 2021

The funnel plot showed that The I2 (variation in ES attributable to heterogeneity) test result showed that there was high heterogeneity with I2 97.5, at a p-value < 0.0001. We also assessed there is no presence of publication bias among studies included in the review. There was an asymmetrical distribution of the included studies through visual inspection, which indicates there was no potential bias (fig.4).

### Factors associated with knowledge towards prevention of covid-19 among pregnant women

The review identified different factors associated with the assessment of knowledge towards the prevention of COVID-19 among pregnant women. Significantly associated factors were age, fear of covid-19, and urban residence. Age was significantly associated with knowledge towards preventing COVID-19 among pregnant women. Using these studies included in a group of meta-analysis assessment of knowledge towards preventive COVID-19 among pregnant women whose age is in the range 20–34 years old is 2.370 times more likely to have the knowledge to prevent COVID-19 (AOR: 2.37, 95% CI: 1.07, 8.236). Pregnant women who fear COVID-19 were 1.408 times more likely to prevent COID-19 than non-fear about COVID-19 (AOR: 1.408, 95% CI: 1.03, 2.019). Another significant factor is urban residence women were 1.838 times more likely to prevent COVID-19 than women who live in a rural area (AOR: 1.838, 95% CI: 1.338, 2.525) (table 2).

**Table 2:** Factors that significantly associated with knowledge towards prevention of covid-19 among pregnant women in a national study in Ethiopia, systemic review 2021

study	ES [95% Conf. Interval]	%Weight	Variables
Fikadu et al (2020)	1.2 (1.15, 22.24)	70.72	Age
Tadess et al (2020)	11.7(1.180,17.80)	29.28	
I- Pooled ES	2.370 (1.07, 8.236)	100	

Tadesse et al	0.130(0.060, 0.310)	19.26	Fear COVID-19
Janakiriam et al	2.485(1.664, 3.711)	80.74	
I-V pooled ES	1.408 (1.03, 2.019)	100.00	

fikadu etal	2.160 (1.240,3.770)	32.59	Urban residence
besh et al	1.700 (1.200,2.600)	67.41	
I-V pooled ES	1.838 (1.338,2.525)	100.00	

## Discussion

Having a good knowledge about COVID-19 is an important mechanism to prevent COVID-19 transmissions. Especially pregnant women should prevent this expanding disease of COVID-19 in the case for themselves and their infants. So that this systemic review and meta-analysis is trying to estimate the pooled prevalence of knowledge and its associated factors towards preventing COVID-19 among pregnant women in Ethiopia.

In this meta-analysis, the pooled prevalence of knowledge towards preventing COVID-19 among pregnant women in Ethiopia was 50.27% (95% CI: 31.60, 68.94). Even though there was no similar meta-analysis study conducted on this specific research questions, the pooled prevalence of assessment of knowledge towards preventing COVID-19 among pregnant women is proportionate to a survey done in the United Arab Emirates 46%^15^ and systemic review in general populations in Ethiopia 40% 16. This study is lower than a study conducted in Iran (85%) 17, India (80.6%) 18, and Ghana (85.6)^19^. This difference might be because of the time of the studies conducted in which information delivery and awareness creation programs about the pandemic are ongoing and improving.

This study shows that younger age pregnant women were more likely knowledgeable as compared to older age pregnant women. This finding is supported by preventive practice in Ethiopia's educated survey finding 20. This explanation might be due to that younger pregnant women high exposed to information and have interested to know about COVID-19 and the younger age also have more educated and most of the time use social media accesses, such as internet, television, and radio to get the information about COVID-19.

In this meta-analysis, pregnant women who fear COVID-19 have 1.4 times more likely to prevent methods of COVID 19 than their counterparts. This finding agrees with those of the studies done in Taiwan^21^ and Iran^22^. The reason might be to have more fear of covid-19 leading to more intention and interest to know about COVID-19, transmission, symptoms, and prevention mechanisms in counter to decrease fear of individuals.

Another factor that towards preventing COVID-19 among pregnant women is urban residence. Pregnant women that live in urban were 1.8 times more likely to know the prevention of COVID 19 than rural residents. This finding is supported by a study in Iran which stated that living in a rural area was associated with poor knowledge among pregnant women^23^. The reason might be pregnant women who live in rural and urban have different access for information, getting from social media like the internet, television health education from health professionals, and radio.

## Conclusion and recommendation

This systemic review showed that only half of the pregnant women in Ethiopia had good knowledge about COVID-19. Age, fear about COVID-19, and urban residence were significantly associated with knowledge towards the prevention of COVID-19 among pregnant women in this review. Health care providers better to be strengthen their awareness creation among rural residents and old-age pregnant women.

## Limitation

The study revealed that had a risk of bias, incomplete retrieval of identified research, and reporting bias were the limitation of the study.

## List of Abbreviations

AOR: Adjusted odds ratio
CS: Cross-sectional
CI: Confidence interval
OR: Odds ratio,
COVID-19: Coronavirus infectious disease

## Declaration

### Availability of data and materials

Full datasets and other materials about this study could be obtained from the corresponding author upon reasonable request.
